# Engineering cyanobacteria to improve photosynthetic production of alka(e)nes

**DOI:** 10.1186/1754-6834-6-69

**Published:** 2013-05-06

**Authors:** Weihua Wang, Xufeng Liu, Xuefeng Lu

**Affiliations:** 1 Key Laboratory of Biofuels, Shandong Provincial Key Laboratory of Energy Genetics, Qingdao Institute of Bioenergy and Bioprocess Technology, Chinese Academy of Sciences, No. 189 Songling Road, Qingdao, 266101, China; 2 University of Chinese Academy of Sciences, Beijing, 100049, China

**Keywords:** Cyanobacteria, *Synechocystis* sp. PCC6803, Alka(e)ne, Fatty acid, Metabolic engineering

## Abstract

**Background:**

Cyanobacteria can utilize solar energy and convert carbon dioxide into biofuel molecules in one single biological system. *Synechocystis* sp. PCC 6803 is a model cyanobacterium for basic and applied research. Alkanes are the major constituents of gasoline, diesel and jet fuels. A two-step alkane biosynthetic pathway was identified in cyanobacteria recently. It opens a door to achieve photosynthetic production of alka(e)nes with high efficiency by genetically engineering cyanobacteria.

**Results:**

A series of *Synechocystis* sp. PCC6803 mutant strains have been constructed and confirmed. Overexpression of both acyl-acyl carrier protein reductase and aldehyde-deformylating oxygenase from several cyanobacteria strains led to a doubled alka(e)ne production. Redirecting the carbon flux to acyl- ACP can provide larger precursor pool for further conversion to alka(e)nes. In combination with the overexpression of alkane biosynthetic genes, alka(e)ne production was significantly improved in these engineered strains. Alka(e)ne content in a *Synechocystis* mutant harboring alkane biosynthetic genes over-expressed in both *slr0168* and *slr1556* gene loci (LX56) was 1.3% of cell dry weight, which was enhanced by 8.3 times compared with wildtype strain (0.14% of cell dry weight) cultivated in shake flasks. Both LX56 mutant and the wildtype strain were cultivated in column photo-bioreactors, and the alka(e)ne production in LX56 mutant was 26 mg/L (1.1% of cell dry weight), which was enhanced by 8 times compared with wildtype strain (0.13% of cell dry weight).

**Conclusions:**

The extent of alka(e)ne production could correlate positively with the expression level of alkane biosynthetic genes. Redirecting the carbon flux to acyl-ACP and overexpressing alkane biosynthetic genes simultaneously can enhance alka(e)ne production in cyanobacteria effectively.

## Background

Interest in engineering cyanobacteria for biofuel production has increased recently driven by using photosynthesis to directly convert carbon dioxide into a desirable fuel [1-6]. Additionally, cyanobacteria exhibit higher solar conversion efficiency and growth rate compared to plants and eukaryotic microalgae [7,8]. *Synechocystis* sp. PCC6803 was the first cyanobacterium for which the complete genome was sequenced in 1996 [9]. So far 126 genomic sequences of cyanobacteria strains are available [10]. Well established genetic manipulation techniques have been applied for cyanobacteria. The techniques make cyanobacteria highly tractable platforms to build efficient biosynthetic pathways for biofuel production by genetic engineering [11].

Alkanes with C4-C23 carbon chain length possess higher energy density, hydrophobic property and compatibility with existing liquid fuel infrastructure, which are the predominant constituents of gasoline, diesel, and jet fuels [12]. They can be produced by various organisms such as bacteria, yeasts, plants and insects [13]. In the late 1960s, production of alka(e)nes was reported in a diversity of cyanobacteria [14]. In 2010, a two-step alkane biosynthetic pathway in cyanobacteria was identified. Acyl-acyl carrier protein (ACP) can be reduced to aldehyde by an acyl-ACP reductase (AAR, EC 1.2.1.50), and then aldehyde can be oxidized to alkane or alkene by an aldehyde-deformylating oxygenase (ADO) [15].

Fatty acid substrates as acyl chains of membrane lipids are biosynthesized by fatty acid synthase (FAS). Acetyl-CoA is converted to malonyl-CoA by a multi-subunit acetyl-CoA carboxylase consisting of AccA, AccB, AccC and AccD, which is the rate-limiting step of fatty acid biosynthesis [16]. Acyl-ACPs synthesized by FAS can be incorporated to membrane lipids. Free fatty acids (FFAs) generated by lipolytic enzymes during degradation of membrane lipids can also be activated to acyl-ACPs by an acyl-ACP synthetase (AAS, EC 6.2.1.20) [17].

Kaczmarzyk and Fulda (2010) established that the only AAS gene in *Synechocystis* sp. PCC 6803 is *slr1609*[17]. The *slr1609*-knockout mutant was incapable of importing exogenous fatty acids and secreted fatty acids released from membrane lipids into the medium. This suggests a remarkable role for this cyanobacterial AAS in recycling released fatty acids [17]. Our previous study showed that the alka(e)ne production was significantly reduced in *slr1609* deletion mutant of *Synechocystis* sp. PCC6803**,** which indicates AAS plays an essential role in alka(e)ne production [18].

**Figure 1:**
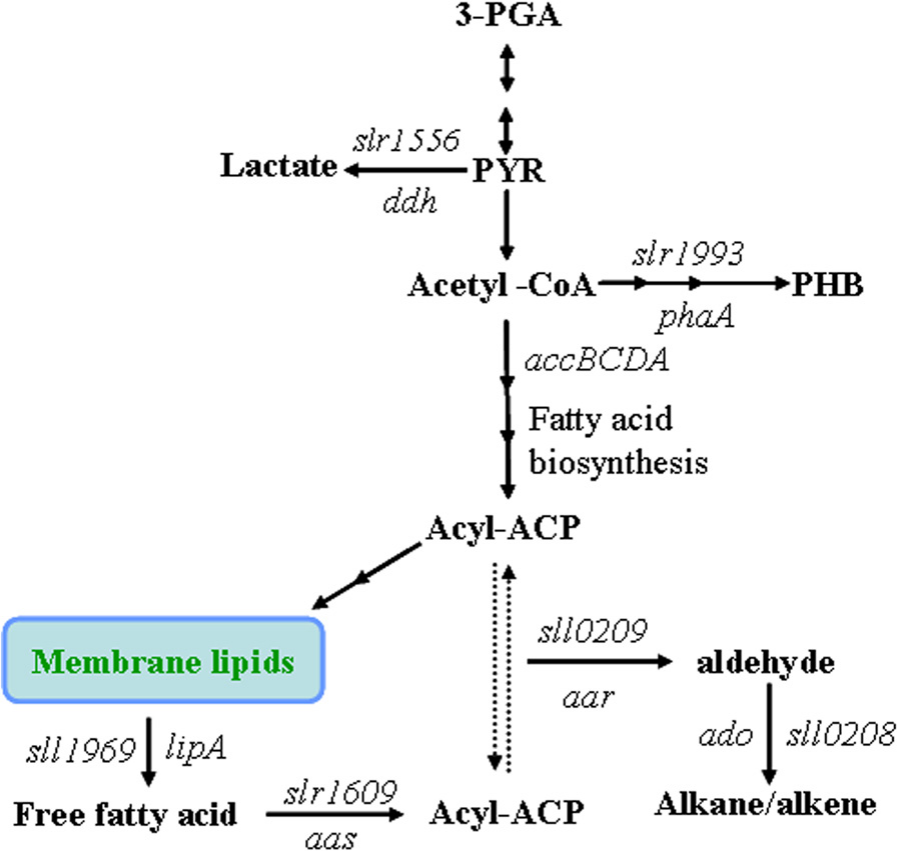
**Schematic overview of fatty acid, alkane (alkene) and main competing metabolic pathways in *Synechocystis *sp. PCC6803.** Key enzyme genes in those pathways are indicated. 3-PGA, glyceraldehyde 3-phosphate; PYR, pyruvate; PHB, poly-β-hydroxybutyrate; acyl-ACP, acyl- acyl carrier protein; *ddh*, 2-hydroxyacid dehydrogenase gene; *phaA*, polyhydroxyalkanoates-specific beta-ketothiolase gene; *accBCDA*, multi-subunit acetyl-CoA carboxylase gene;*lipA*, lipolytic enzyme gene; *aas*, acyl-ACP synthetase gene; *aar*, acyl-ACP reductase gene; *ado*, aldehyde-deformylating oxygenase.

Alka(e)ne biosynthesis was reported in a diversity of cyanobacteria [19]. Heptadecane and heptadecene are the major constituents of alka(e)nes in *Synechocystis* sp. PCC6803**,** and the total alka(e)ne content was about 0.1% of the cell dry weight (DW) [5,20]. Alkane biosynthetic genes from cyanobacteria were heterologously expressed in *Escherichia coli* and *Synechococcus* sp. PCC 7002, and alka(e)ne production ranged from 5–40 mg/L in *E. coli* and reached 5% of DW in *Synechococcus* sp. PCC 7002 [13,21]. Redirecting the glyceraldehyde 3-phosphate (3-PGA) originated from Calvin-Benson-Bassham cycle to acyl-ACP and enhancing the expression of alkane biosynthetic genes should improve efficiency and yield of alka(e)ne production in *Synechocystis* sp. PCC6803 (Figure 1).

In this study, metabolic engineering approaches were employed to construct a series of *Synechocystis* sp. PCC6803 mutant strains. Alka(e)ne production was enhanced by 8.3 times in one of these modified strains by overexpressing alkane biosynthetic genes in two different loci of the genome.

## Results and discussion

### Alka(e)ne production can be doubled in *Synechocystis* mutants overexpressing cyanobacteria alkane biosynthetic genes

**Figure 2:**
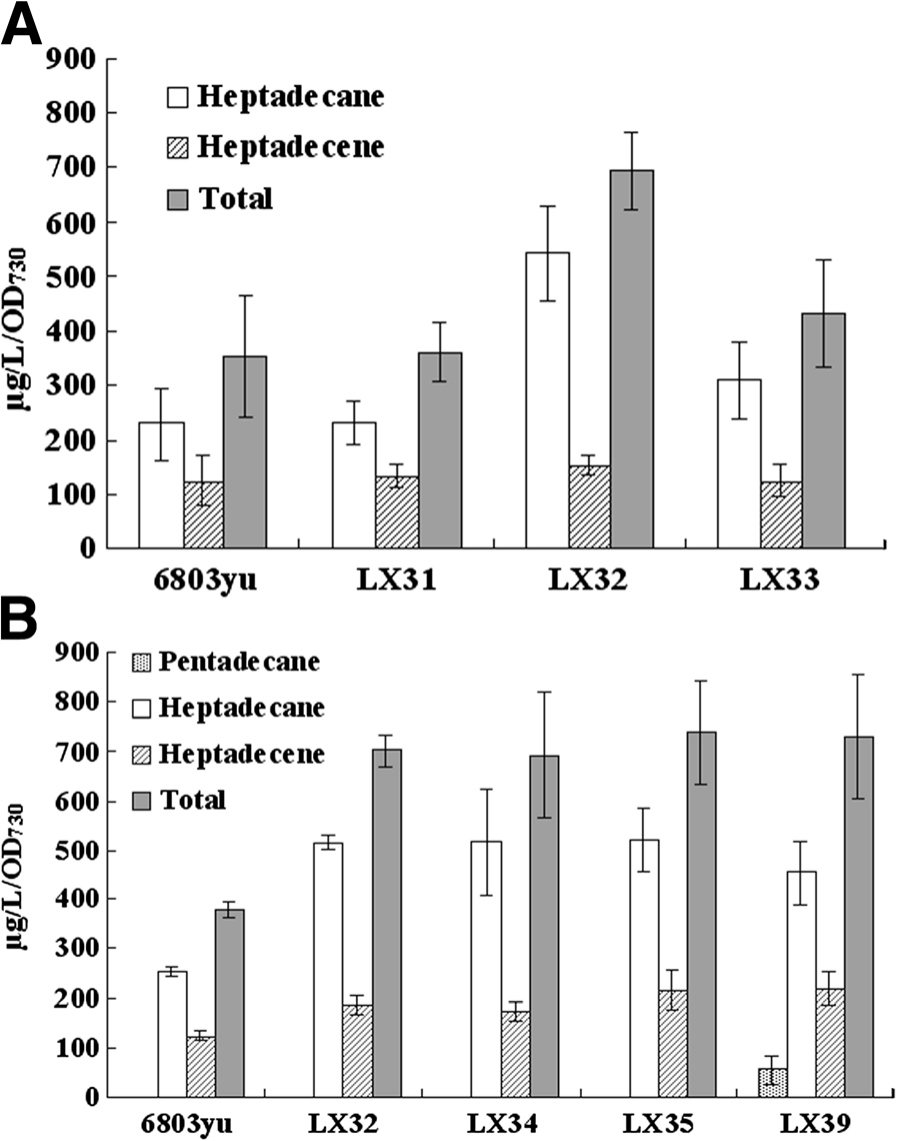
**Alka(e)ne production in *Synechocystis *mutants overexpressing cyanobacteria alkane biosynthetic genes.** (**A**) Alka(e)ne production in *Synechocystis* mutants overexpressing *sll0208* (LX31), *sll0209* (LX33) and both genes (LX32) compared with *Synechocystis* sp. PCC6803 (6803yu). Error bars represent the standard deviation of three replicates. (**B**) Alka(e)ne production in *Synechocystis* mutants overexpressing *orf1594* and *Npun_R1711* (LX39), alkane biosynthetic genes from *Synechococcus elongatus* PCC7942 (LX34) and *Nostoc punctiforme* PCC73102 (LX35) compared with wildtype strain (6803yu) and LX32 mutant. Error bars represent the standard deviation of three replicates.

*Synechocystis* mutants overexpressing either or both native alkane biosynthetic genes (*sll0208* and *sll0209*) were constructed. The production of alka(e)ne can be doubled in the mutant overexpressing both *sll0208* and *sll0209* (LX32, about 700 μg/L/OD) compared with parent strain (6803yu, about 300 μg/L/OD), and no significant changes was detected for the mutant strain only expressing either *sll0208* (LX31) or *sll0209* (LX33) as shown in Figure 2A.

Alkane biosynthetic genes from *Synechococcus elongatus* PCC7942 (*orf1593* and *orf1594*) and *Nostoc punctiforme* PCC73102 (*npun1710* and *npun 1711*) were also overexpressed in *Synechocystis* sp. PCC6803. Alka(e)ne production can also be doubled in these two mutants (LX34 and LX35, Figure 2B). These results indicate that enhanced activity of AAR and ADO resulting from overexpression can convert more acy-ACP available to alka(e)ne.

Pentadecane can be produced in *Synechococcus elongatus* PCC7942, while no pentadecane was detected in the *Synechocystis* mutant overexpessing *orf1593* and *orf1594* (LX34). The *in vitro* enzyme activity assays performed by Eser *et al.* (2011) suggested that the *Nostoc punctiforme* PCC73102 ADO may possess higher activity than the *Synechocystis* sp. PCC6803 ADO [22]. The highest reported titers of alka(e)nes from this pathway involved *E. coli* overexpressing *orf1594* (*aar*) and *Npun_R1711* (*ado*) among 16 different combinations of the two enzymes from a variety of cyanobacteria [13]. In the *Synechocystis* mutant overexpressing *orf1594* and *Npun_R1711* (LX39), alka(e)ne production was not improved significantly compared with other three mutants (LX32, LX34 and LX35), while about 60 μg/L/OD_730_ pentadecane can be produced in this mutant (Figure 2B).

### **Redirecting the carbon flux to acyl-ACP can enhance** alka(e)ne **production in cyanobacteria effectively**

**Figure 3:**
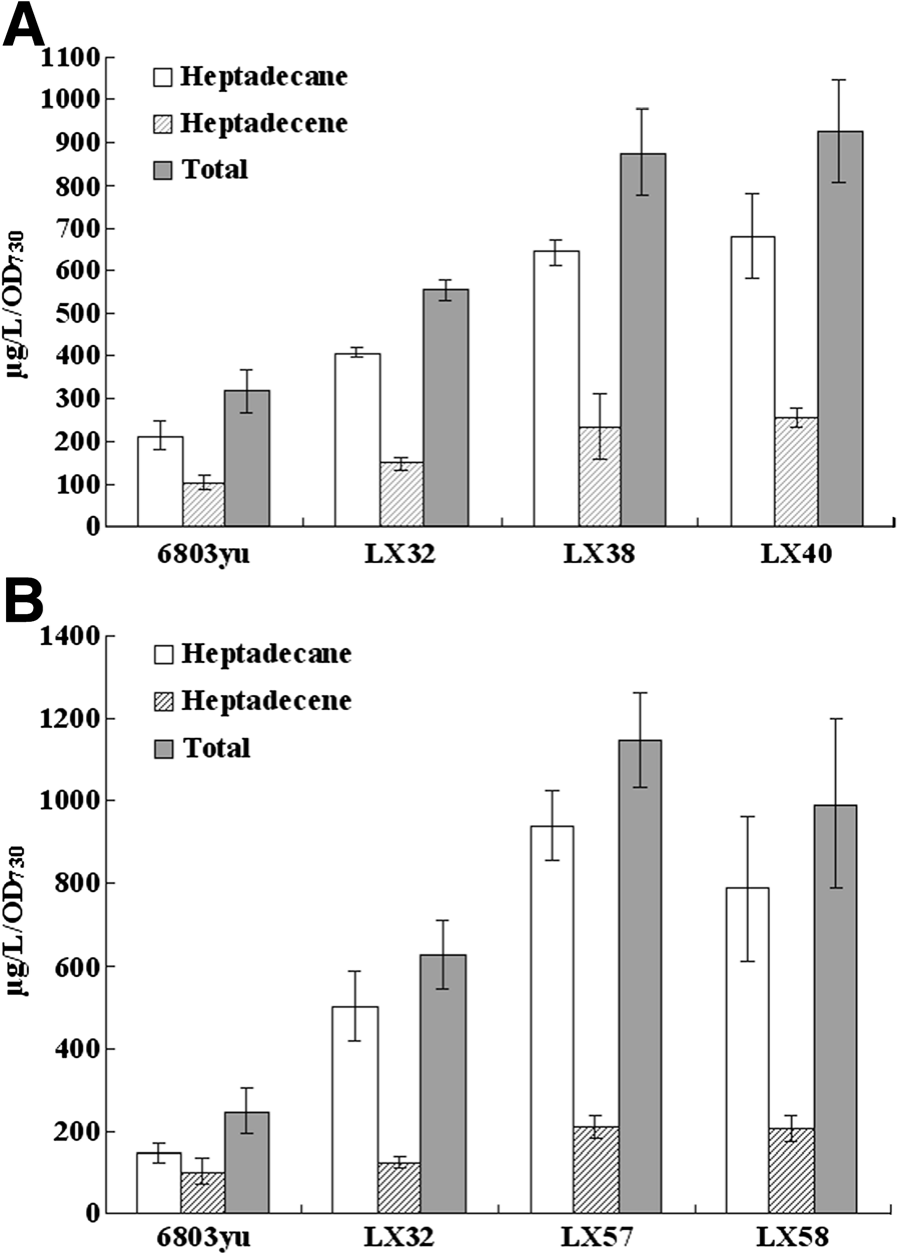
**Alka(e)ne production can be enhanced effectively by redirecting the carbon flux to acyl-ACP.** (**A**) Alka(e)ne production in *Synechocystis* mutants overexpressing *sll0208 and sll0209* in *slr1609* over-producing strain (LX38) and *phaA* gene deletion mutant (LX40) compared with wildtype strain (6803yu) and LX32 mutant. Error bars represent the standard deviation of three replicates. (**B**) Alka(e)ne production in *Synechocystis* mutants overexpressing *sll0208 and sll0209* in acetyl-CoA carboxylase genes (LX57) and lipolytic enzyme gene (LX58) over-producing strain compared with wildtype strain (6803yu) and LX32 mutant. Error bars represent the standard deviation of three replicates.

Since acyl-ACP is the immediate substrate for alka(e)ne biosynthesis, redirecting the carbon flux to acyl-ACP may enhance downstream alka(e)ne production in cyanobacteria [20]. AAS plays an essential role in recycling the released fatty acids to acyl-ACP [17]. Our previous work showed that native alka(e)ne production was not enhanced by overexpressing *slr1609* alone. Maybe activities of AAR and ADO are too low to convert acyl-ACP to alka(e)ne efficiently [18]. A *Synechocystis* mutant overexpressing alkane biosynthetic genes and *slr1609* (LX38) showed enhanced productivity of alka(e)ne by 130% and 60% when compared to *Synechocystis* sp. PCC6803 and LX32 mutant, respectively (Figure 3A). Overexpressing AAS, AAR and ADO gene simultaneously may improve acyl-ACP pool and downstream alka(e)ne production. Considering our previous observation of a 90% reduction of alka(e)ne content in *slr1609* deletion mutant [18], acyl-ACPs from FFA activation by AAS may be predominant source of alka(e)ne in *Synechocystis* strains.

*Synechocystis* sp. PCC6803 can accumulate poly-β-hydroxybutyrate (PHB) as carbon and energy storage compound [23]. Acetyl-CoA and NADPH are required for PHB synthesis. The β-ketothiolase encoded by *phaA* (*slr1993*) condenses two molecules of acetyl-CoA to acetoacetyl-CoA, which is the first step of PHB biosynthesis. Alka(e)ne profiles of the *Synechocystis* mutant overexpressing *sll0208 and sll0209* with *slr1993* deletion (LX40) was analyzed, and the alka(e)ne productivity was enhanced by 150% and 70% compared with *Synechocystis* sp. PCC6803 and LX32 mutant, respectively (Figure 3A). Overexpression of AAR and ADO gene and deletion of PHB biosynthetic gene(s) simultaneously can divert acetyl-CoA and NADPH into production of fatty acid and enhance production of fatty acid-derived alka(e)nes.

Acyl-ACP pool may also be improved by increasing activity of acetyl-CoA carboxylase (ACC), which is the bottleneck of fatty acid biosynthesis. In a *Synechocystis* mutant overexpressing *accBCDA* genes from our previous work, a 56% increase of native alka(e)ne production was obtained [5]. With this mutant, the *sll0208* and *sll0209* were overexpressed to yield LX57 strain. The alka(e)ne production of LX57 mutant was enhanced by 3.6 times when compared to *Synechocystis* sp. PCC6803 (Figure 3B).

The lipolytic enzymes are capable of hydrolyzing acyl chains from membrane lipids. FFAs released from membrane lipids can be activated to acyl-ACPs by an AAS. Cyanobacteria have plenty of membrane lipids and a dynamic lipid metabolism. Based on sequence identity analysis, *sll1969* was annotated as a putative lipolytic enzyme gene *(lipA*). Liu and Curtiss (2012) showed that less FFAs were released in *Synechocystis sll1969*-deletion mutant than the wildtype strain [24]. Alka(e)ne productivity of the *Synechocystis* mutant overexpressing *sll0208, sll0209* and *sll1969* (LX58) was enhanced by 3 times in comparison with wildtype strain (Figure 3B). Increasing activities of LipA can degrade more membrane lipids into FFAs. FFA can be activated to acyl-ACP by AAS. Since acyl-ACP is the immediate substrate for alka(e)ne biosynthesis, overexpressing *sll1969* would lead to an increased alka(e)ne biosynthesis.

### Alka(e)ne production can be improved significantly in *Synechocystis* mutants overexpressing multiple copies of alkane biosynthetic genes

**Figure 4:**
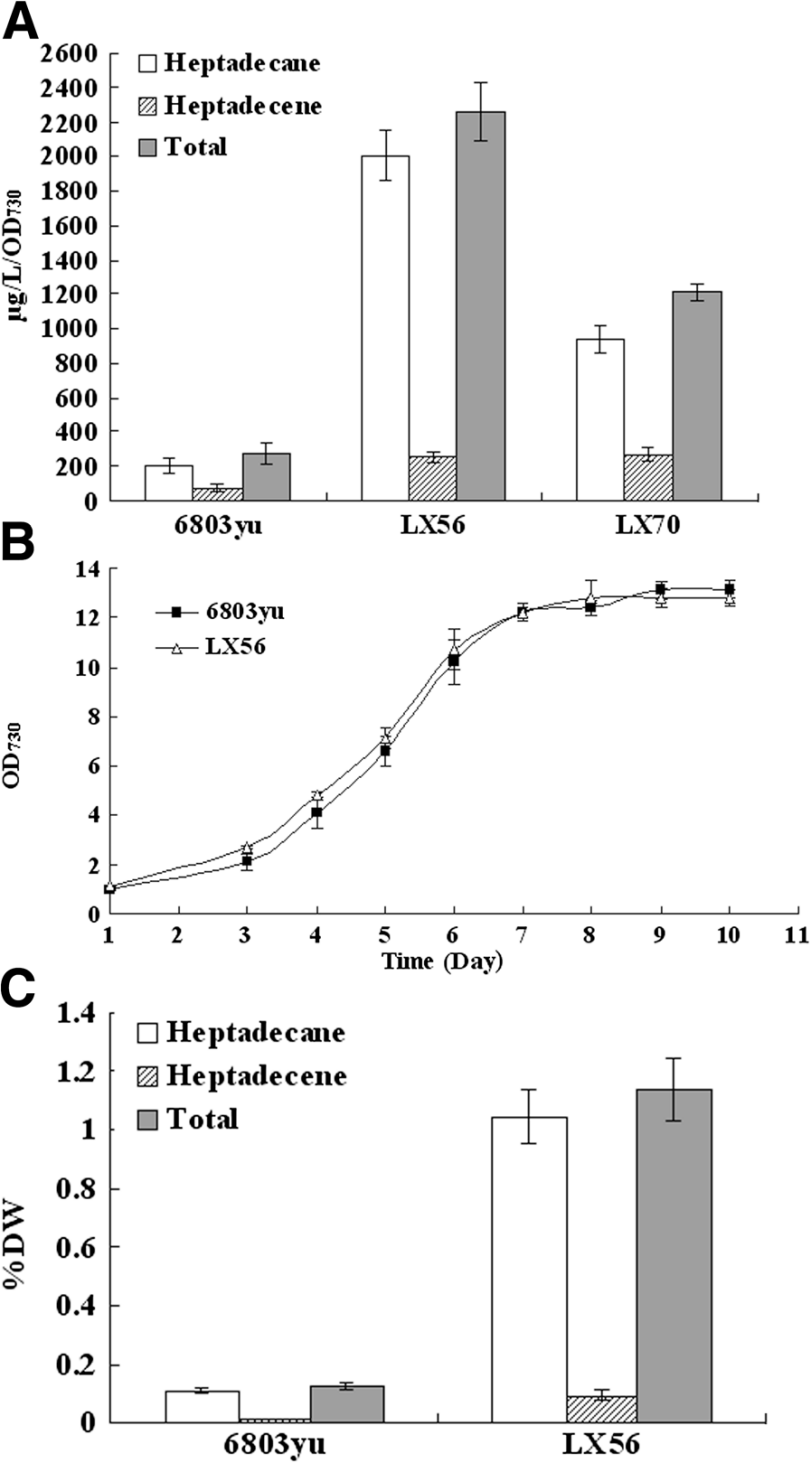
**Alka(e)ne production in *Synechocystis *mutants overexpressing two copies of alkane biosynthetic genes.** (**A**) Alka(e)ne production in the *Synechocystis* mutant overexpressing *sll0208 and sll0209* in both *slr0168* and *ddh* gene sites (LX56) compared with wildtype strain (6803yu) and the *Synechocystis* mutant overexpressing two copies of *sll0208 and sll0209* in *slr0168* site (LX70). Error bars represent the standard deviation of three replicates. (**B**) Growth curves of wildtype strain and the LX56 strain in the bubble column photo-bioreactors. Error bars represent the standard deviation of three replicates. (**C**) Alka(e)ne production calculated as a percentage of DW of LX56 strain was enhanced by 8 times compared with wildtype strain when cultivated in the bubble column photo-bioreactors. Error bars represent the standard deviation of three replicates.

To investigate whether alka(e)ne productivity could be further enhanced by overexpressing multiple copies of alkane biosynthetic genes, *Synechocystis* mutants overexpressing two copies of *sll0208 and sll0209* were constructed. Alka(e)ne productivity of a *Synechocystis* mutant overexpressing two copies of *sll0208* and *sll0209* in *slr0168* site (LX70) was 1.2 mg/L/OD_730_ (Figure 4A). Alka(e)ne productivity of LX56 strain overexpressing *sll0208 and sll0209* in both *slr0168* and *slr1556* (2-hydroxyacid dehydrogenase gene, *ddh*) loci was 2.3 mg/L/OD_730_ (Figure 4A). The final DW of wildtype and LX56 strain in shake flasks was 0.44 and 0.5 g/L, respectively. Alka(e)ne production of wildtype strain in shake flasks was 0.14% of DW (0.64 mg/L). Alka(e)ne production of LX56 strain was enhanced by 8.3 times, up to 1.3% of DW (6.5 mg/L).

**Figure 5:**
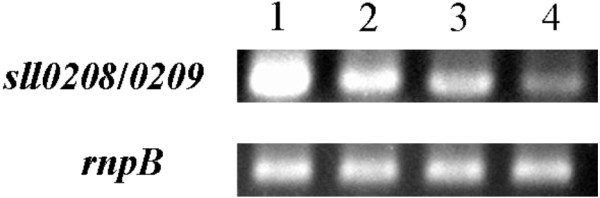
**Semi-quantitative reverse transcription PCR analysis of the transcriptional levels of *sll0208 and sll0209 *in wildtype, LX32, LX70 and LX56 mutant.** The *rnpB* gene was used as the external standards. Lane 1, LX56; Lane 2, LX70; Lane 3, LX32; Lane 4: wildtype.

The transcriptional levels of *sll0208 and sll0209* were steadily increased in wildtype, LX32, LX70 and LX56 mutant in semi-quantitative reverse transcription PCR analysis (Figure 5), which indicated alka(e)ne production could correlate positively with the expression of alkane biosynthetic genes to some extent. Transcription of two adjacent copies of *sll0208 and sll0209* may interfere with each other, so transcription level of two copies of *sll0208 and sll0209* in tandem in LX70 is lower than that of two copies of *sll0208 and sll0209* in separate gene locus in LX56. Overexpressing alkane biosynthetic genes in multiple gene loci can significantly improve the efficiency of alka(e)ne production in cyanobacteria. Similar effects can also be found when multiple-site overexpression was applied to cyanobacteria ethanol or ethylene production [6,25].

Small-scale photo-bioreactors for cultivation of cyanobacteria are required for precise characterization of wildtype and engineered strains to optimize the culture conditions and alka(e)ne production. The LX56 mutant and wildtype strain exhibited similar growth characteristics when cultivated in the bubble column photo-bioreactors. Both of the cultures in photo-bioreactors reached a much higher density (OD_730_~13) than cultures in shake flasks (OD_730_ ~3 to 4) at stationary phase (Figure 4B), and the final DW of wildtype and LX56 strain in photo-bioreactors was 2.2 and 2.3 g/L, respectively. The alka(e)ne production of wildtype strain was 2.8 mg/L, which was 0.13% of DW. The alka(e)ne production of LX56 strain was enhanced by 8 times compared with the wildtype strain, up to 26 mg/L, which was 1.1% of DW (Figure 4C).

In the previous reports, alka(e)ne production ranged from 5 to 40 mg/L in *E. coli*[13] and reached 5% of DW in *Synechococcus* sp. PCC 7002 [21] by heterologously expressing cyanobacterial AAR and ADO genes. Integrating different strategies of genetic modifications used in these studies into a *Synechocystis* mutant strain will further enhance yield of alka(e)nes. Medium chain alkane is less toxic to cyanobacteria than alcohol, acid and other non-native products [26], which leaves huge room for engineering cyanobacteria to improve alka(e)ne production.

## Conclusions

Overexpressing cyanobacteria alkane biosynthetic genes and redirecting the carbon flux to acyl-ACP can improve alka(e)ne production in cyanobacteria significantly. The results indicate that metabolic engineering strategies are powerful for engineering cyanobacteria to overproduce alka(e)nes. Low activities of AAR and ADO need to be enhanced by protein engineering for further improvement of alka(e)ne production. It is also important to understand the physiological roles and regulatory mechanism of native alka(e)nes in cyanobacterial cell.

## Methods

### Chemicals and reagents

Eicosane was purchased from Sigma-Aldrich (USA). Other chemicals were from Merck (Germany) or Ameresco (USA). Oligonucleotides and gene synthesis were carried out by Sangon (Shanghai, China). Taq DNA polymerase and all restriction endonucleases were obtained from Fermentas (Canada) or Takara (Japan). The DNA ladders were from Takara (Japan). The kits used for molecular cloning were from Omega (USA) or Takara (Japan).

### Plasmid construction

Plasmids constructed and used in this study were listed in Table 1.

**Table 1:** Plasmids constructed and used in this study

**Plasmid**	**Relevant characteristics**^**a, b**^	**Reference**
pFQ9R	Ap^r^ Spe^r^, pKW1188sl derivative containing *Omega*, T_rbc_ terminator, P_rbc_ promoter	[5]
pXT37b	Ap^r^, Spe^r^, pUC9' derivative containing upstream and downstream fragments of *slr1609*, *Omega* and P_petE_ promoter.	[5]
pXT119	Ap^r^, pMD18-T derivative containing upstream and downstream fragments of *slr1556*	This study
pLX1	Ap^r^ Spe^r^, pFQ9R derivative containing *sll0208* gene, P_rbc_ promoter	This study
pLX3	Ap^r^ Spe^r^, pFQ9R derivative containing *sll0208* and *sll0209* gene, P_rbc_ promoter	This study
pLX4	Ap^r^ Spe^r^, pXT37b derivative containing *sll0209* gene, P_petE_ promoter	This study
pLX5	Ap^r^ Spe^r^, pFQ9R derivative containing *orf1593* and *orf1594* gene, P_rbc_ promoter	This study
pLX6	Ap^r^ Spe^r^, pFQ9R derivative containing *npun1710* and *npun 1711* gene, P_rbc_ promoter	This study
pLX9	Ap^r^ Spe^r^, pFQ9R derivative containing *orf1594* and *npun 1711* gene, P_rbc_ promoter	This study
pLX13	Ap^r^, Kan^r^, pXT119 derivative containing *sll0208* and *sll0209* gene*, CK2, and* P_rbc_ promoter*.*	This study
pLX14	Ap^r^, pGEM-T Easy derivative containing *sll0208* and *sll0209* gene, P_rbc_ promoter.	This study
pLX27	Ap^r^, pGEM-T Easy derivative containing *sll0208* and *sll0209* gene, P_rbc_ promoter, T_rbc_ terminator.	This study
pLX28	Ap^r^, Kan^r^, pGEM-T Easy derivative containing *sll0208* and *sll0209* gene, P_rbc_ promoter, T_rbc_ terminator, *CK2*.	This study
pLX59	Ap^r^ Spe^r^, pFQ9R derivative containing two fragments of P_rbc_ promoter *, sll0208* and *sll0209* gene.	This study

^a^ Ap, Ampicillin. Spe, Spectinomycin. Kan, kanamycin.

^b^*CK2* for kanamycin antibiotic resistance gene and *Omega* for spectinomycin antibiotic resistance gene.

*E. coli* strain DH5a was used for molecular cloning. *Synechocystis* sp*.* PCC6803 and *Synechococcus elongatus* PCC7942 were generous gifts from Prof. Xudong Xu of Institute of Hydrobiology, Chinese Academy of Sciences. *Nostoc punctiforme* PCC73102 was a generous gift from Prof. John C. Meeks of UC Davis, USA.

Alkane biosynthetic genes *sll0208* along with *sll0209* were amplified from the genomic DNA of *Synechocystis* sp. PCC6803 with the primers 020809F1/020809R1. The primers 0208F1/0208R1 and 0209F1/0209R1 were used to amplify *sll0208* and *sll0209* gene respectively. Alkane biosynthetic genes *orf1593* along with *orf1594* were amplified from the genomic DNA of *Synechococcus elongatus* PCC7942 with the primers 9394F1/9394R1. The primers 1594F1/1594R1 were used to amplify *orf1594* gene. Alkane biosynthetic genes *npun1710* and *npun1711* were amplified from the genomic DNA of *Nostoc punctiforme* PCC73102 with the primers 1011F1/1011R1. The primers 1711F1/1711R1 were used to amplify *npun1711*. The *sll0208* gene, *sll0208* along with *sll0209*, *orf1593* along with *orf1594*, *npun1710* along with *npun1711*, *orf1594* along with *npun1711* were subcloned into *Xba*I*/Sma*I site of the plasmid pFQ9R [5], resulting in pLX1, pLX3, pLX5, pLX6, pLX9 plasmid, respectively. The *sll0209* gene was subcloned into *Nde*I*/Xho*I site of the plasmid pXT37b [5] to generate plasmid pLX4.

The *ddh* gene were amplified from the genomic DNA of *Synechocystis* sp PCC6803 by PCR using the primers ddh-F/ddh-R and inserted into the TA cloning site of pMD18-T-Simple, to generate the plasmids pXT119. The plasmid pLX3 was used as the template to amplify the 2.5 kb fragment of *P*_*rbc*_ promoter , *sll0208* and *sll0209* and Rubisco terminator (*T*_*rbc*_) [5] using the primers rbcNF/rbcNR. The 2.5 kb fragment was inserted into the TA cloning site of pGEM-T Easy to generate the plasmids pLX27. The *ck2* cassette was excised with *Sal*I and *Xba*I from pRL446 [27] and inserted into the *Sal*I/*Spe*I site of pLX27, to generate the plasmid pLX28. The 3.6kb fragment containing *ck*2, *P*_*rbc*_*, T*_*rbc*_, *sll0208* and *sll0209* was digested with *Nde*I and *Sph*I from pLX28 and cloned into *Bgl*II site of pXT119 with blunt ends, to generate the plasmid pLX13.

The *P*_*rbc*_ promoter and *sll0208* along with *sll0209* were amplified from the plasmid pLX3 by fusion PCR using the primers PrbcBX-F/PrbcK-R, 0809K-F/0809B-R. The above fragment with *Xba*I site and *Spe*I/*Sal*I sites on the 5’ and 3’ ends respectively was inserted into the TA cloning site of pMD19-T-Simple to generate the plasmids pLX14. To utilize the isocaudarner pair *Xba*I and *Spe*I, the fragment containing two copies of *P*_*rbc*_, *sll0208* and *sll0209* gene in tandem was digested with *Xba*I and *Sal*I, and cloned into *Xba*I/*Sal*I site of pFQ9R, to generate the plasmid pLX59. Plasmid maps were listed in Additional file 1: Figure S1.

### Transformation and construction of *Synechocystis* sp. PCC6803 mutant strains

Strains constructed and used in this study were listed in Table 2.

**Table 2:** ***Synechocystis *strains constructed and used in this study**

**Strain**	**Genotype**^**a,**^	**Reference**
6803yu	*Synechocystis* sp. PCC6803 Wild-type, Glucose-tolerance	Prof. Xudong Xu
XT203	*slr1993*∷*CK2 sacB*	This lab
GQ4	*psbA2*∷*CK2 P*_psbA2_*slr1609*	This lab
GQ10	*slr0168*∷Omega *P*_rbc_*sll1969 T*_rbc_	This lab
Syn-20ACC	*slr0168::*Omega *P*_*rbcl*_*accBCDA* (PCC6803) *Trbc*	[5]
LX31	*slr0168*∷Omega *P*_rbc_*sll0208 T*_rbc_	This study
LX32	*slr0168*∷Omega *P*_rbc_*sll0208&sll0209 T*_rbc_	This study
LX33	*slr0168*∷Omega P_petE_*sll0209*	This study
LX34	*slr0168*∷Omega *P*_rbc_*orf1593&orf1594 T*_rbc_	This study
LX35	*slr0168*∷Omega *npun1710&npun 1711 T*_rbc_	This study
LX38	*slr0168*∷Omega *P*_rbc_*sll0208&sll0209T*_rbc_, *psbA2*∷*CK2 P*_psbA2_*slr1609*	This study
LX39	*slr0168*∷Omega *orf1594&npun 1711 T*_rbc_	This study
LX40	*slr0168*∷Omega *P*_rbc_*sll0208&sll0209 T*_rbc_, *slr1993*∷*CK2 sacB*	This study
LX55	*slr1556*∷*CK2 P*_rbc_*sll0208&sll0209 T*_rbc_	This study
LX56	*slr0168*∷Omega *P*_rbc_*sll0208&sll0209 T*_rbc_, *slr1556*∷*CK2 P*_rbc_*sll0208&sll0209 T*_rbc_	This study
LX57	*slr1556*∷*CK2 P*_rbc_*sll0208&sll0209 T*_rbc_, *slr0168::*Omega *Prbcl accBCDA* (PCC6803) *Trbc*	This study
LX58	*slr1556*∷*CK2 P*_rbc_*sll0208&sll0209 T*_rbc_, *slr0168*∷Omega *P*_rbc_*sll1969 T*_rbc_	This study
LX70	*slr0168*∷Omega *P*_rbc_*sll0208&sll0209 P*_rbc_*sll0208&sll0209*	This study

^*a*^*P*_petE_, 0.4 kb DNA fragment containing the promoter of *petE* gene. *P*_rbc_, 0.3 kb DNA fragment containing the promoter of *rbc* operon. *P*_*rbcl*_*,* 1.3 kb DNA fragment containing the promoter of *rbc* operon. *T*_rbc_, 0.2 kb downstream DNA fragment of *rbcS* gene. *P*_psbA2_, 1.5 kb DNA fragment containing the promoter of *psbA2* gene. All promoters and terminators mentioned here are from *Synechocystis* sp. PCC 6803.

All of the above plasmids were checked by enzyme digestion and then transformed to *Synechocystis* cells. The transformations of *Synechocystis* strains with plasmids were performed as described [28]. The plasmids pLX1, pLX3, pLX4, pLX5, pLX6, pLX9, pLX13 and pLX59 were transformed to *Synechocystis* sp. PCC6803 to generate the mutant strains LX31, LX32, LX33, LX34, LX35, LX39, LX55 and LX70, respectively. The plasmid pLX3 was transformed to *Synechocystis* mutant strain XT203 and GQ4 to generate LX40 and LX38 mutant strains, respectively. The plasmid pLX13 was transformed to *Synechocystis* mutant strain GQ10, Syn-20ACC and LX32 to generate LX58, LX57, and LX56 mutant strains, respectively. For the initial selection of transformants, the DNA/cell mixture was applied to BG11 agar plates. After 18 h the membrane filters were applied to fresh BG11 agar plates containing following antibiotics (10μg mL^-1^ spectinomycin, 10 μg mL^-1^ erythromycin or 5 μg mL^-1^ spectinomycin/kanamycin). Homogeneous mutants were obtained by successive streaking on BG11 plates with appropriate antibiotics. Homologous integration of the expressing cassette and complete segregation were confirmed by PCR using primers listed in Additional file 1: Table S1. Schematic diagrams for homogeneous recombination of different plasmids were listed in Additional file 1: Figure S2. PCR analysis of the genotype of *Synechocystis* mutant strains were displayed in Additional file 1: Figure S3.

### Cultivation of *Synechocystis* strains

Normal liquid cultures of all *Synechocystis* strains in this study were grown at 30°C in 500 mL shake flasks containing 300 mL BG11 medium with aeration by sterile air under constant illumination at a photosynthetic photon flux density of approximately 30 μmol photons m^-2^ s^-1^ of white light. When necessary, the following antibiotics were added: kanamycin (20 μg mL^-1^) and spectinomycin (20 μg mL^-1^)_._ Cell growth of each culture was monitored by measuring OD_730_. *Synechocystis* sp. PCC6803 wildtype and the mutant strains exhibited similar growth rate and final cell density. The cultures cultivated in shake flasks were harvested at OD_730_ of 3 to 4 after 14 days, when the stationary phase reached. All *Synechocystis* strains in this study were cultivated in shake flasks first to evaluate yields of alka(e)nes. The alka(e)ne content of LX56 mutant with the highest alka(e)ne yield in this work and the wildtype control cultivated in shake flasks were calculated as a percentage of DW. Conversion between OD_730_ and DW of LX56 mutant and *Synechocystis* sp. PCC6803 cultivated in shake flasks was performed by regression analysis (Additional file 1: Figure S4).

The bubble column photo-bioreactor was a 580 mm×30 mm glass column with a silica gel plug. *Synechocystis* sp. PCC6803 and the LX56 mutant strain were grown in flasks to exponential phase and harvested by centrifugation. The harvested cells were re-suspended in 200 mL fresh BG11 media, and transferred to the column photobioreactors at 30°C under 50 μEm^-2^ s^-1^ of white light with air bubbling for 24h, after which the light intensity was adjusted to 100 μEm^-2^ s^-1^ and the aeration was switched to 5% (v/v) CO_2_-enriched air. LX56 mutant was grown in the presence of 10 μg mL^-1^ kanamycin and 10 μg mL^-1^ spectinomycin_._ Cell growth of *Synechocystis* sp. PCC6803 and LX56 mutant was monitored by measuring OD_730_.

### **Extraction and GC-MS analysis of** alka(e)nes

Alka(e)nes were extracted from *Synechocystis* cells. 200 mL normal culture or 50 mL culture of column photo-bioreactor at stationary phase was harvested by centrifugation. The cells were resuspended in 10 mL of TE buffer (pH8.0) and then lysed by sonication. 50 μL eciosane (1 mg/mL) was added to the cell lysate as the internal standard for alka(e)ne analysis. The lysate was extracted for 1h at room temperature with 10 mL chloroform–methanol (v/v, 2:1) [29]. A two-phase system (top: aqueous, bottom: organic) was generated after shaking for 1 h and centrifugation at 8000 rpm at room temperature for 15 min. The bottom organic phase was transferred to a new glass tube and evaporated to dryness under a stream of nitrogen at 55°C. The residue was dissolved in 1mL of n-hexane. Aliquots of this mixture were analyzed by GC-MS using an Agilent 7890A-5975C system equipped with a HP-INNOWax (30 m×250 μm×0.25 μm). Helium (constant flow 1 mL/min) was used as the carrier gas. The temperature of the injector was 250°C and the following temperature program was applied: 100°C for 1 min, increase of 5°C min^-1^ to 150°C then increase of 10°C min^-1^ to 250°C for 15 min. The internal standard was used to determine alka(e)ne yield, which was reported as the mean based on three independent experiments.

### Semi-quantitative reverse transcription PCR

RNA was isolated from 50 ml cultures of *Synechocystis* cells in mid-exponential phase by using TRIzol Reagent (Life Technologies). The first-strand cDNA was synthesized from 1μg of total RNA using a RevertAid First Strand cDNA synthesis Kit (Thermo SCIENTIFIC) according to the manufacturer’s protocol. PCR was performed using primers 0809RTF1/R1 (Additional file 1: Table S1) to amplify 350 bp of internal coding region of *sll0208 and sll0209*. The RNase P subunit B (*rnpB*) gene-specific primer pairs rnpB1/2 (Additional file 1: Table S1) were designed to amplify *rnpB* as external standards. Thirty cycles were used for *rnpB* cDNA, and 35 cycles were used for *sll0208 and sll0209* cDNA.

## Abbreviations

ACP: Acyl carrier protein; AAR: Acyl-ACP reductase; ADO: Aldehyde-deformylating oxygenase; ACS: Acyl-CoA synthetase; AAS: Acyl-ACP synthetase; ACC: Acetyl-CoA carboxylase; FAS: Fatty acid synthase; FFA: Free fatty acid; DW: Cell dry weight; PCR: Polymerase chain reaction; OD: Optical density.

## Competing interests

The authors declare that they have no competing interests.

## Authors’ contributions

XL (Xuefeng Lu) conceived of the study. XL (Xuefeng Lu), WW and XL (Xufeng Liu) designed the experiments. WW and XL (Xufeng Liu) carried out experiments including the construction and cultivation of *Synechocystis* sp. PCC683 mutant strains, extraction and analysis of alka(e)nes, and GC-MS analysis. XL (Xuefeng Lu), WW and XL (Xufeng Liu) wrote the manuscript. All authors read and approved the final manuscript.

## Supplementary Material

Additional file 1: Table S1 Primers used in this study. **Figure S1.** Plasmid maps. **Figure S2.** Schematic diagrams for homogeneous recombination of different plasmids. **Figure S3.** PCR analysis of the genotype of *Synechocystis* mutant strains. **Figure S4.** Linear regression of cell dry weight (DW) versus OD_730_ for *Synechocystis* sp. PCC6803(6803yu)and LX56 strain cultivated in shake flasks. Click here for file
