# Supplementary material for: Engineering cyanobacteria to improve photosynthetic production of alka(e)nes
Source: Biotechnol Biofuels. 2013 May 6;6:69. doi: 10.1186/1754-6834-6-69 (PMC3679977; doi:10.1186/1754-6834-6-69)
Supplement: Additional file 1: Table S1 — Primers used in this study. Figure S1. Plasmid maps. Figure S2. Schematic diagrams for homogeneous recombination of different plasmids. Figure S3. PCR analysis of the genotype of Synechocystis mutant strains. Figure S4. Linear regression of cell dry weight (DW) versus OD730 for Synechocystis sp. PCC6803(6803yu)and LX56 strain cultivated in shake flasks. [file 1754-6834-6-69-S1.doc]

**Table S1. Primers used in this study.**

| **primer** | **sequence** | **reference** |
| --- | --- | --- |
| 0208F1 | 5'TCTAGACCCGAGCTTGCTGTCCGC3' | This study |
| 0208R1 | 5'CCCGGGCCACAGATCCTAAGAAGC3' | This study |
| 0209IF1 | 5'CATATGATGTTTGGTCTTATTGGTC3' | This study |
| 0209IR1 | 5'CTCGAGGCCTAAAGAGCTACTAAAG3' | This study |
| 020809F1 | 5'TCTAGACCCGAGCTTGCTGTCCGC3' | This study |
| 020809R1 | 5'CCCGGGTCACCAGCTCCACACACG3' | This study |
| 9394F1 | 5'TCTAGAATGCCGCAGCTTGAAGCCA3' | This study |
| 9394R1 | 5'CCCGGGTCAAATTGCCAATGCCAAG3' | This study |
| 1011F1 | 5'TCTAGAATGCAGCAGCTTACAGACC3' | This study |
| 1011R1 | 5'CCCGGGTTTGTCCTTTGTCCTTTGC3' | This study |
| 1594F1 | 5'TCTAGAATGTTCGGTCTTATCGGTC 3' | This study |
| 1594R1 | 5'CCCGGGTCAAATTGCCAATGCCAAG3' | This study |
| 1711F1 | 5'TCTAGAATGCAGCAGCTTACAGACC3' | This study |
| 1711R1 | 5'CCCGGGCAGCACTCAGCACTCGTTA3' | This study |
| rbcNF | 5' CCATGGCTCACCATTTGGACAAAACA3' | This study |
| rbcNR | 5' CCATGGCCCACTTAGATAAAAAATCC3' | This study |
| 0168-1 | 5'ACCTCTCCACGCTGAATTAG3' | [5] |
| 0168-2 | 5'TTCCAGGCCACATTGTTGTC3' | [5] |
| pD1-2d-2 | 5'TCCACACTGGGAAGTTTGCC3' | [15] |
| pD1-3 | 5'AGCTTCGTGTATATTAACTTCCTGT3' | [15] |
| Pha-S | 5'GGGGACCATCCTGACTACACGG3' | This study |
| phaAB-4 | 5'TGTTGATGGTGGGTATCGTGGTG3' | This study |
| Ck2-t | 5'GATCTTGCCATCCTATGGAACTG3' | This study |
| ddh-F | 5'CAGAGGAGTTTGTCATAGGAGC3' | This study |
| ddh-R | 5'TACCCATTTCAACCTCAACGAT3' | This study |
| PrbcBX-F | 5'AGATCTTCTAGACCATTTGGACAAAACATCAGGA3' | This study |
| PrbcK-R | 5'CGGTGCGGACAGCAAGCTCGGGCATGGTACCCTAGGTCAGTCCTCCATAAACATTG3' | This study |
| 0809K-F | 5'CAATGTTTATGGAGGACTGACCTAGGGTACCATGCCCGAGCTTGCTGTCCGCACCG3' | This study |
| 0809B-R | 5'GGATCCGGCCTAAAGAGCTACTAAAGGG3' | This study |
| 0809RTF1 | 5GTAACATCGGCTTCAGCACC'3' | This study |
| 0809RTR1 | 5'AATCTTCAGCAACCGCTTGG3' | This study |
| rnpB-1 | 5'GTTAGGGAGGGAGTTGCGG3' | This study |
| rnpB-2 | 5'AAGAGAGTTAGTCGTAAGCCG3' | This study |

**Figure S1**


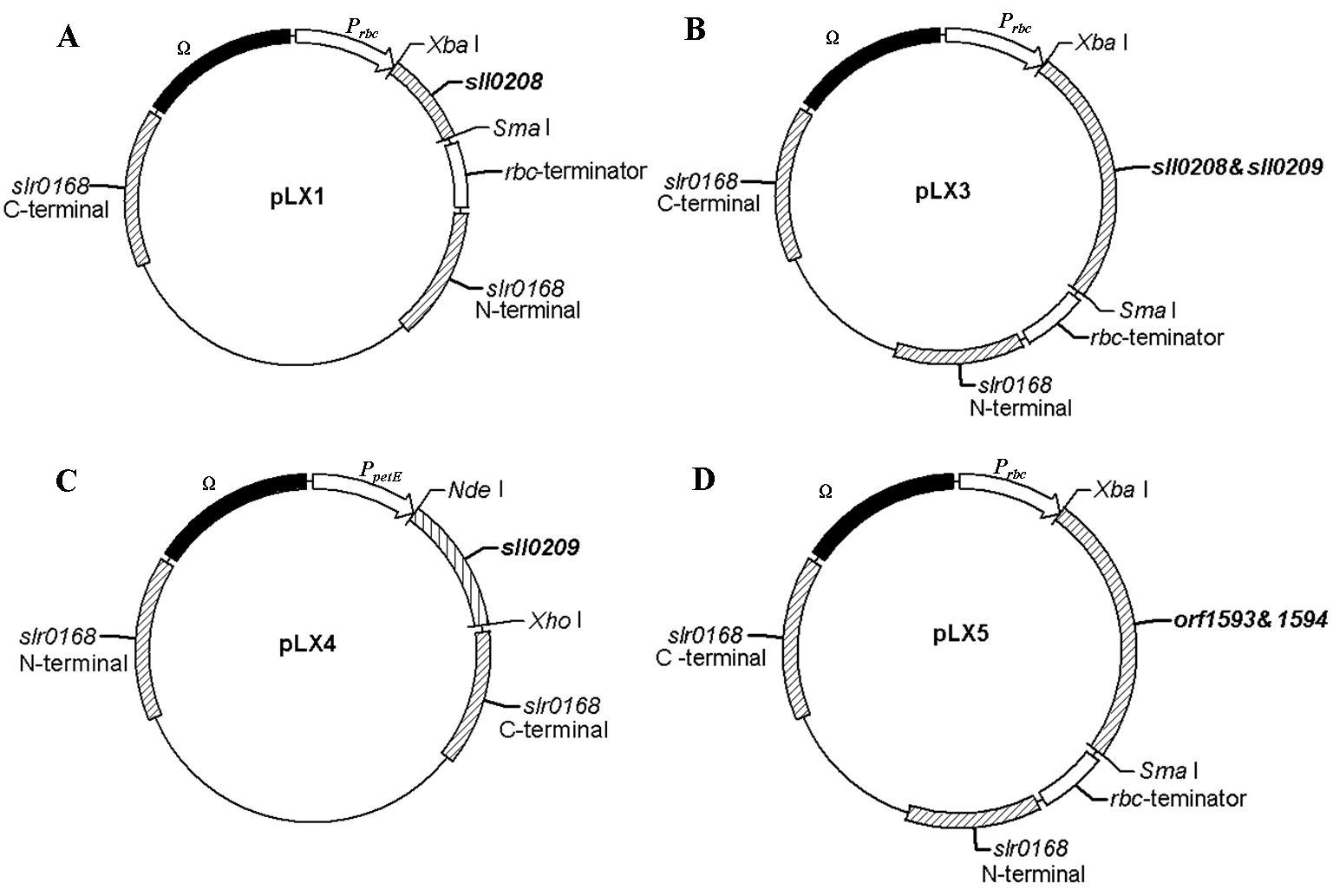


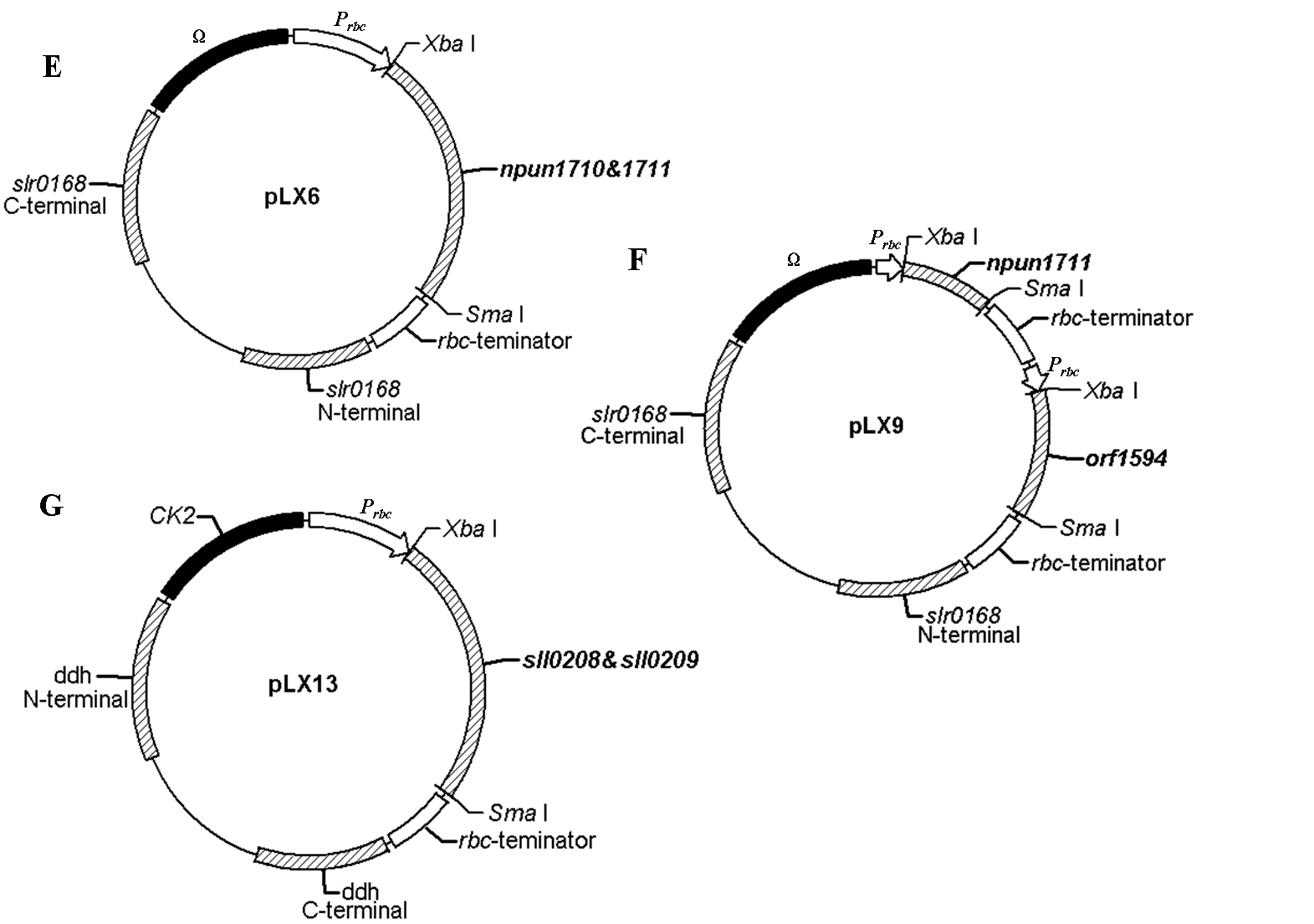


**Figure S1.** Plasmid maps

**Figure S2**


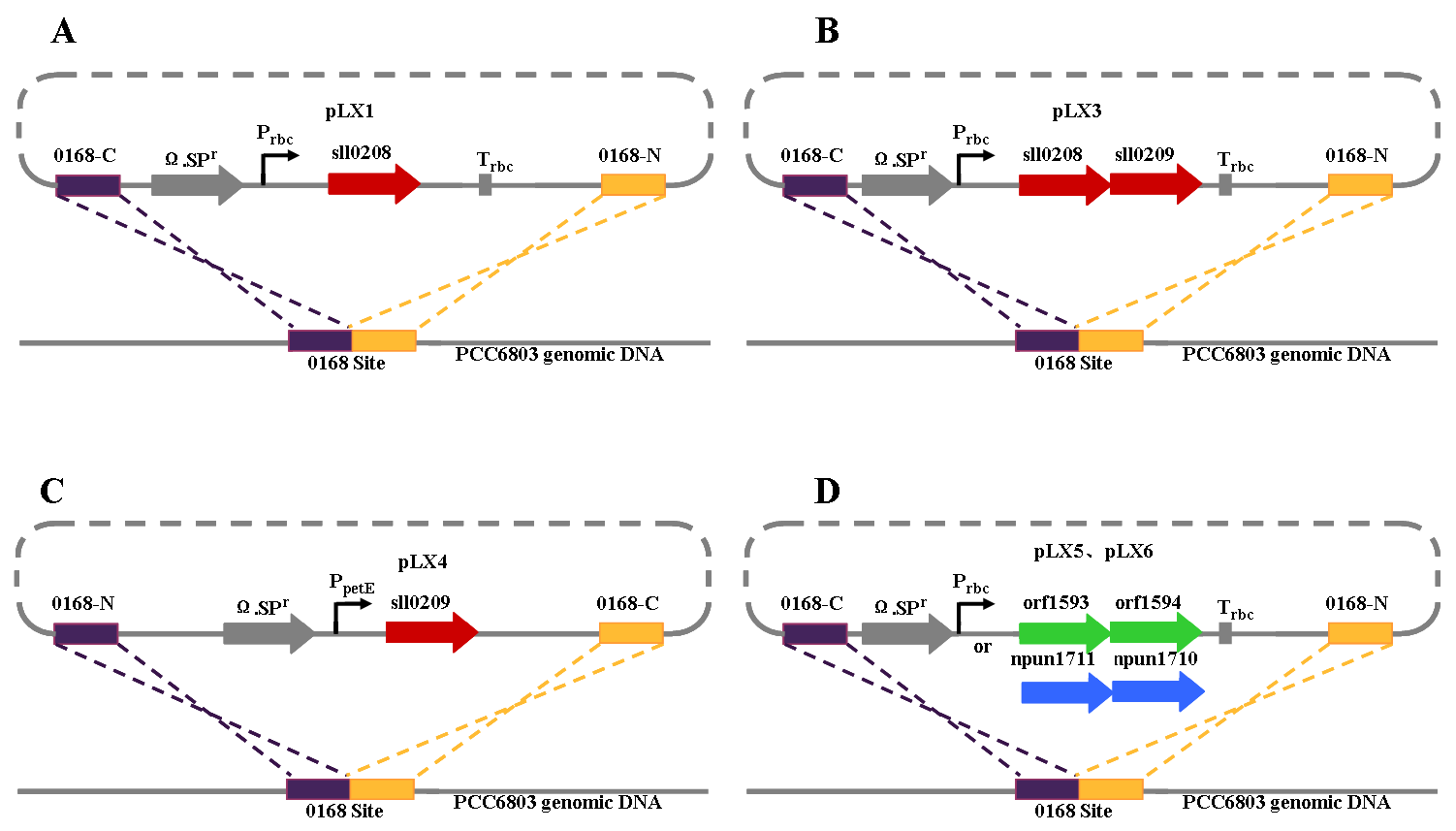


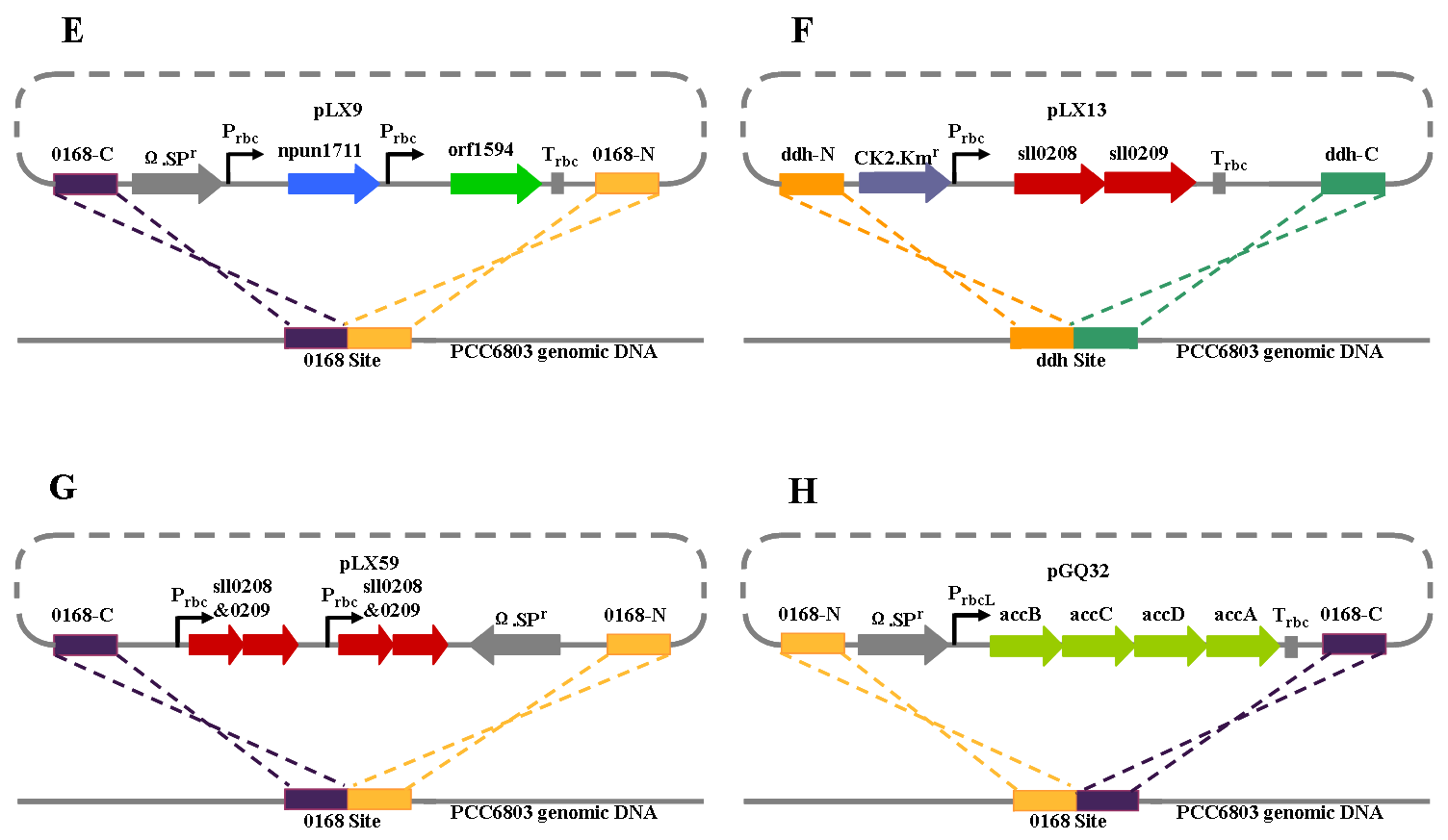


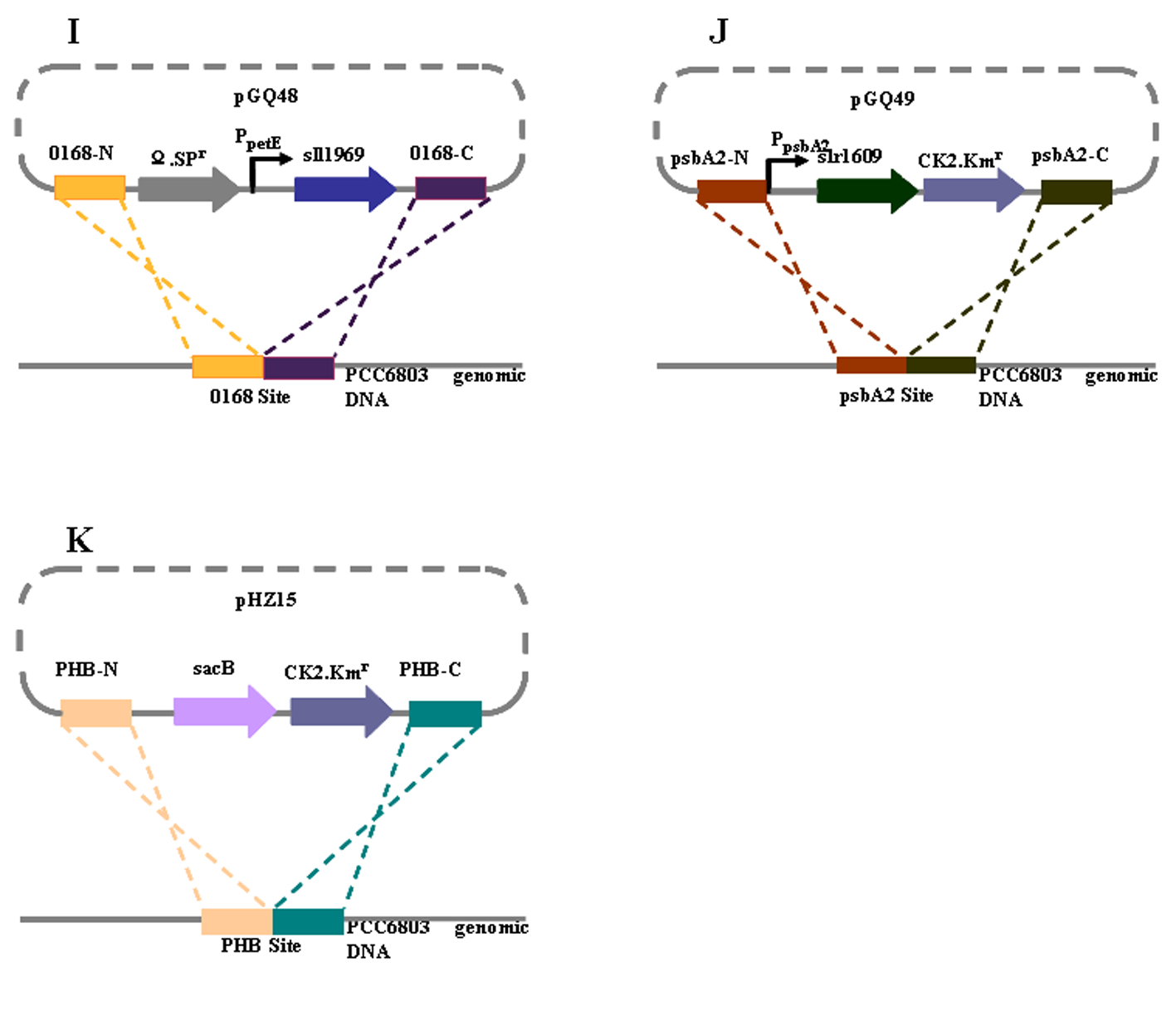


**Figure S2.** Schematic diagrams for homogeneous recombination of different plasmids

**Figure S3**

**
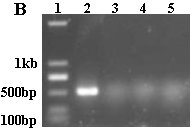

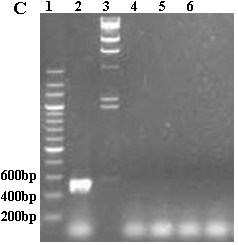

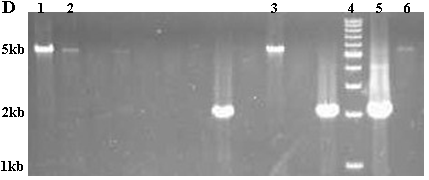

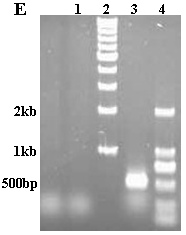
**

**
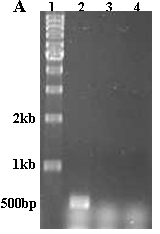
**

**Figure S3. PCR analysis of the genotype of *Synechocystis* mutant strains.**

**(A)** **lane 1:** DNA marker (1kb DNA Ladder Marker), **lane 2:** genomic DNA of wild-type was amplified by primers 0168-1 and 0168-2 (control), **lane 3:** genomic DNA of LX31 was amplified by primers 0168-1 and 0168-2, **lane 4:** genomic DNA of LX33 was amplified by primers 0168-1 and 0168-2.

**(B) lane 1:** DNA marker (DL2000 DNA Ladder Marker), **lane 2:** genomic DNA of wild-type was amplified by primers 0168-1 and 0168-2 (control), **lane 3:** genomic DNA of LX32 was amplified by primers 0168-1 and 0168-2, **lane 4:** genomic DNA of LX34 was amplified by primers 0168-1 and 0168-2, **lane 5:** genomic DNA of LX35 was amplified by primers 0168-1 and 0168-2.

**(C) lane 1:** DNA marker (200bp DNA Ladder Marker), **lane 2:** genomic DNA of wild-type was amplified by primers 0168-1 and 0168-2 (control), **lane 3:** DNA marker (λHindIII DNA Ladder Marker), **lane 4:** genomic DNA of LX38 was amplified by primers 0168-1 and 0168-2, **lane 5:** genomic DNA of LX39 was amplified by primers 0168-1 and 0168-2, **lane 6:** genomic DNA of LX40 was amplified by primers 0168-1 and 0168-2.

**(D)** **lane 1:** genomic DNA of LX58 was amplified by primers ddh-F and ddh-R, **lane 2:** genomic DNA of LX57 was amplified by primers ddh-F and ddh-R, **lane 3:** genomic DNA of LX56 was amplified by primers ddh-F and ddh-R, **lane 4:** DNA marker (1kb DNA Ladder Marker), **lane 5:** genomic DNA of wild-type was amplified by primers ddh-F and ddh-R (control), **lane 6:** genomic DNA of LX55 was amplified by primers ddh-F and ddh-R.

**(E) lane 1:** genomic DNA of LX70 was amplified by primers 0168-1 and 0168-2, **lane 2:** DNA marker (1kb DNA Ladder Marker), **lane 3:** genomic DNA of wild-type was amplified by primers 0168-1 and 0168-2 (control), **lane 4:** DNA marker (DL2000 DNA Ladder Marker).

**Figure S4**

**Figure S4.**  Linear regression of cell dry weight (DW) versus OD_730_ for *Synechocystis* sp. PCC6803（6803yu）and LX56 strain cultivated in shake flasks
